# Factors Influencing Use of Fitness Apps by Adults under Influence of COVID-19

**DOI:** 10.3390/ijerph192315460

**Published:** 2022-11-22

**Authors:** Yanlong Guo, Xueqing Ma, Denghang Chen, Han Zhang

**Affiliations:** 1 Social Innovation Design Research Centre, Anhui University, Hefei 203106, China; 2 Anhui Institute of Contemporary Studies, Anhui Academy of Social Sciences, Hefei 203106, China; 3 Department of Science and Technology Communication, University of Science and Technology of China, Hefei 203106, China; 4 Research Center for Science Communication, Chinese Academy of Sciences, Hefei 203106, China; 5 College of Environmental Science and Engineering, Ocean University of China, Qingdao 266100, China

**Keywords:** COVID-19, adult group, fitness APP, influencing factors

## Abstract

During the coronavirus disease 2019 (COVID-19) pandemic, many countries imposed restrictions and quarantines on the population, which led to a decrease in people’s physical activity (PA) and severely damaged their mental health. As a result, people engaged in fitness activities with the help of fitness apps, which improved their resistance to the virus and reduced the occurrence of psychological problems, such as anxiety and depression. However, the churn rate of fitness apps is high. As such, our purpose in this study was to analyze the factors that influence the use of fitness apps by adults aged 18–65 years in the context of COVID-19, with the aim of contributing to the analysis of mobile fitness user behavior and related product design practices. We constructed a decision target program model using the analytic hierarchy process (AHP), and we analyzed and inductively screened 11 evaluation indicators, which we combined with an indicator design questionnaire. We distributed 420 questionnaires; of the respondents, 347 knew about or used fitness apps. Among these 347, we recovered 310 valid questionnaires after removing invalid questionnaires with a short completion time, for an effective questionnaire recovery rate of 89.33%. We used the AHP and entropy method to calculate and evaluate the weight coefficient of each influencing factor and to determine an influencing factor index. Our conclusions were as follows: first, the effect of perceived usefulness on the use of fitness apps by the study groups was the most notable. Second, personal motivation and perceived ease of use considerably influenced the adult group’s willingness to use fitness apps. Finally, the perceived cost had relatively little effect on the use of fitness apps by adults, and the study group was much more concerned with the privacy cost than the expense cost.

## 1. Introduction

The COVID-19 pandemic has hugely impacted people’s ways of living, intellectual health, and quality of life worldwide [1]. The imposition of lockdown and quarantine measures on populations has been used to restrict the spread of COVID-19, but such measures have also had many serious consequences [2]. According to the results of multi-country surveys, measures such as restraint and seclusion have negatively impacted social participation, lifestyle pleasure, mental health, psychosocial and emotional disorders, sleep quality, and employment status [3,4,5]. Some authorities announced a stoppage of all services and activities except for a few basic services, which led to necessary adjustments in the lifestyles of the affected populations, which severely damaged their mental health. This was manifested by increased stress in the general population and an increase in the number of depressions [6,7]. These abrupt modifications in people’s lives included, among others, physical activity and exercise. Ammer et al. stated that home confinement during COVID-19 led to a reduction in physical activity (PA) and an increase of approximately 28% in the time spent sitting each day [8].

How humans coped and found approaches to being physically healthy in the face of pandemic-related restrictions (home isolation and closed gyms, parks, and gymnasiums) needs to be understood. Through health apps, users changed their traditional method of engaging in fitness imposed by time and geographical barriers and could choose to exercise anytime and anywhere, record their physical condition, and more flexibly control their exercise. People’s intention to use fitness apps has substantially increased. However, the churn rate of these apps is high, with over 45% of customers stopping after the novelty wears off, so an in-depth perception of consumer motivation and the elements influencing the use of health apps is required [9,10]. To gain insight into these issues, in this study, we collected user data using a questionnaire and constructed a decision-goal scenario model based on TAM through the analytic hierarchy process (AHP). The questionnaire included questions about the user’s basic information and what factors affected their use of fitness apps. We analyzed the user data to assess the factors that affected their continued use of fitness apps. Next, we reviewed the literature on the impact of the pandemic on physical health and described the factors influencing the use of fitness apps, and then presented the details of our analysis and the final findings.

## 2. Literature Review

Sports and physical exercise play a vital role in the physical and intellectual health of an individual [11]. The U.S. Physical Activity Guidelines suggest that all adults, even those with chronic conditions, should engage in at least 150 to 300 min of moderate-intensity exercise per week if they are capable [12]. Haider stated that decreased PA levels may negatively affect fitness and can be related to an increase in nervousness and despair [13]. The findings of a study in Austria showed an increase in the duration of predominant depressive signs and symptoms from 3% to 6% between pre- and post-pandemic [14]. Harleen et al. conducted semi-structured smartphone interviews in 2020 with 22 adults who had usually exercised at a fitness center before the COVID-19 pandemic but who stayed at home at some point during the countrywide lockdown. The results of the analysis showed that participants’ situational perceptions at some stage during the lockdown were extremely negative, and they lacked the motivation to exercise at a gym. They exhibited mental health concerns and an over-reliance on social media. However, performing general health exercises indoors during lockdown remarkably helped them to overcome their psychological problems and fitness issues [15].

While experiencing a forced adaptation to new norms of maintaining social distancing, health apps can assist humans to manipulate a change in their dietary intake, engaging in both healthy and bodily activity, and promoting a wholesome lifestyle [16]. Based on the above advantages, humans from all groups seized the opportunities provided by the commercial online health industry, which vigorously improved their offerings of online fitness. This situation actively promoted the digital reform of the ordinary health industry. The Talking Data 2014 Mobile Internet Data Report showed that the number of users of mobile health management on both iOS and Android platforms reached 120 million, which was an increase of 113.4% from January to December 2014, and the growth rate was increasing. Users of apps such as Goudong and Le Power Running have exceeded 10 million in number, and the number of downloads of Nike Training and Super Diet King has increased by more than 300%. Sports and fitness apps have a wide range of people using them. In addition, the use of sports and health apps to assist in guiding exercise will change traditional sports and fitness methods, creating a shift in digital and scientific fitness.

In the context of the rapid development of mobile fitness apps, many scholars have focused on the factors influencing their use, engaging in theoretical research and practical studies. When studying the factors influencing college students’ fitness app use, Yi considered fitness motivation, leisure, entertainment motivation, and structure rationality hardware requirements as antecedent variables of the perceived ease of use (PEOU) and perceived usefulness (PU) based on the technology acceptance model (TAM). Yi considered the perceived value variables as direct elements influencing university students’ mindset toward health app use [17]. The empirical findings showed that PEOU, PU, and perceived price positively affected college students’ attitudes toward using mobile fitness apps. PEOU was positively influenced by the ease of operation experienced when using the health software program and the rationality of the health software program design, whereas perceived price was influenced by cellular hardware requirement, the cost of the software program, and the value obtained in the course of its use. The factors that positively influenced PU were, in descending order, fitness motivation, PEOU, motivation to acquire fitness knowledge, perceived cost, and motivation to record fitness activities [18]. Cui investigated the willingness to use mHealth programs based on the technology readiness and acceptance model (TRAM) and extended the model by introducing health awareness. The constructed model was tested by surveying 639 mobile fitness app users and potential users using AMOS 22.0. The test results showed that optimism, revolutionary spirit, and health perception were necessary antecedent variables for the PEOU and PU of cell phone health apps, which indirectly influenced the intention to use. PU and usage mindset directly influenced cell phone health app users’ intention to use them [19,20,21,22]. Ardion et al. conducted a technology acceptance model (TAM) test considering trust, social influence, and health valuation on 476 German fitness app users, examining the factors influencing the German users’ intentions to continue using specific fitness software. The outcomes of the structural equation modeling showed that the respondents’ intention to use a particular health app was primarily based on three factors: PEOU, PU, and prohibitive social norms [23].

In summary, in the context of the COVID-19 pandemic, country-wide fitness awareness has increased, and mobile phone fitness app use has become a commonly accepted new form of exercise [24]. Nowadays, the user and industry scales of mobile fitness apps are rapidly growing, and the mobile fitness industry has broad market prospects and is now an emerging area of general interest in the industry. In this context, we selected fitness apps as the research object and analyzed which factors affected the use of fitness apps through theoretical analysis and empirical testing. Our results benefit the analysis of mobile fitness user behavior and related product design practices.

## 3. Research Methodology

In this study, we first reviewed a large amount of the literature to determine the content of the study. We then summarized the relevant literature about the theoretical knowledge of technology acceptance, perceived cost, and self-determination theory and analyzed the relationship between them. Next, we selected reasonable judgment indicators to provide a theoretical basis for the subsequent study [25]. In determining the study population, according to the 2021 United Nations World Health Organization, the classification of age groups placed those aged 0–17, 18–65, 66–79, 80–99, and 100 years or more into the categories of minors, adults, middle-aged people, elderly people, and long-lived people, respectively. Among them, those who should pay the most attention to physical exercise and have a strong ability to make independent choices are adults aged 18–65 years. Therefore, in this study, we distributed a questionnaire to the study group and collected the data from the questionnaires. We screened the initially recovered data and then used SPSSAU for reliability and validity analysis. Finally, we used two assignment methods, AHP, and entropy weighting, to derive the comprehensive weighting results and analyze the relevant indicators affecting the weighting of the use of fitness apps by adults under 65 years old [26].

### 3.1. Hierarchical Analysis and Entropy Method

We needed to analyze the factors affecting the use of fitness apps by adults from multiple dimensions, and we selected the AHP method, which is used to combine the qualitative and quantitative aspects of multi-objective complex problems to calculate the decision weights and use the experience of decision makers to judge the relative importance of the weights between the criteria of whether each measurement goal can be achieved. The entropy weighting method is combined with the resynthesis of indicator weights to assign values, and the use of comprehensive weights makes the results more scientific, fair, and persuasive.

### 3.2. Indicator Construction

In our analysis of the factors influencing the use of fitness apps by adults aged 18–65 years, we needed to consider the current pandemic and policy guidance, the characteristics of health app use, and the relevant research results to build a scientific and reasonable indicator system. A wide range of elements may influence health app use: they may be multilevel, multifactor, and multi-indicator. For evaluation index selection, by collecting the opinions of relevant experts and designers, our final hierarchy of the fitness app-use influencing factors included one target layer, four guideline layers, and eleven program layers.

#### 3.2.1. Establishing Guideline Level Indicators

The technology acceptance model (TAM), proposed by Davis et al. in 1989, is one of the most influential theories in the field of information systems research. In the preliminary TAM, PU, and PEOU are the elements that directly impact the usage attitude and user behavior through attitude intention [27]. Davis et al. reported that PEOU refers to the effort customers perceive as being required to operate a new technology; PU refers to how many customers accept as true that the technological device will enhance their overall work performance [27]. Karah anna et al. demonstrated that PEOU and PU affect the users’ use behavior, and PEOU additionally impacts PU [28]. Bildad et al. found that the ease of using Internet technology plays a key role in improving user faith in software builders [29]. Therefore, in the specific construction of the corresponding indicators, we used PEOU (B1) and PU (B2) [30,31].

**Figure 1:**
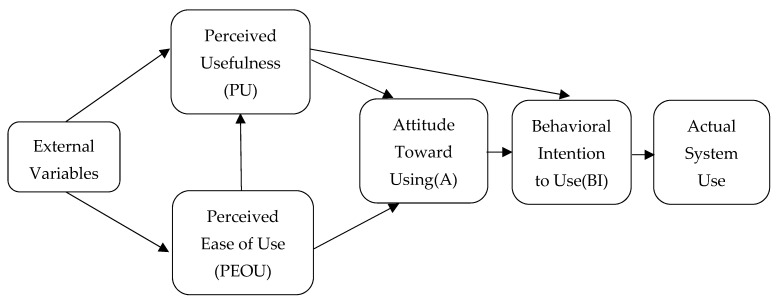
Technology acceptance model.

Despite the broad applicability of the TAM (Figure 1), the model can be modified by adding external premises and theoretically sound elements, which can expand the predictive power of the model [32]. The self-determination principle has been widely used to help encourage physical activity in individuals, and intrinsic motivation represents an archetype of independent activity, where people are motivated by intrinsic motivation and are free to engage in activities independent of external factors [33,34,35]. According to self-determination theory, consumer motivation (the reason why a person engages in an activity) and consumer-aim (the purpose for this activity) is intently associated [36]. In the field of advertising and customer behavior studies, researchers typically agree that customers perceive the cost as a necessary factor influencing purchase decisions: and the greater the perceived cost-utility of a product, the greater the motivation to buy it [37]. Regarding the elements affecting the perceived value, most researchers have considered the antecedent variables of the perceived cost for empirical analysis. Perceived immediate use advantages (i.e., perceived gains) and perceived sacrifices (i.e., perceived losses) are the antecedent variables of perceived cost [38]. Some scholars have also used factors such as perceived risk, cost of purchase, quality of service, and the quality of the product as antecedent variables affecting the consumers’ perceived value (Wood and Scheer, 1996; Zhong, K., 2013) [39]. Regarding the elements impacting the customer’s perceived value, we introduced two achievable variables to the technology acceptance model: perceived cost (B3) and personal motivation (B4) [40].

#### 3.2.2. Determination of Program-Level Indicators

We analyzed and inductively screened 11 evaluation indicators from H1 to H11 according to the detailed division of the elements used for evaluating the first-level indicators (Table 1). To measure indicator B1 (PU), we used the scale developed by Yang et al. to set three measurement indicators: content adaptability (H1), content relevance (H2), and content quality (H3) [41]. To measure indicator B2 (PEOU), we used the scale developed by Gong et al.: the technology level (H4), interaction effectiveness (H5), and system compatibility (H6) [42,43,44,45]. For B3, the perceived value indicator, we measured the financial cost (H7) and privacy cost (H8) based totally on the evaluation by San et al., who focused on the effects of the perceived advantages and perceived dangers of people’s transactional conduct [46,47,48]. To measure B4 (personal motivation indicator), we applied the scale developed by Park et al. and set three measures: health concerns (H9), outcome expectations (H10), and social influence (H11) [49,50,51].

### 3.3. Questionnaire Design

Based on the literature review of the effect of the pandemic and health apps, we chose eleven attributes to examine the factors influencing the use of health apps amongst adults aged 18–65 years to determine the impact of COVID-19. We assessed these 11 attributes with a questionnaire (Table 2). We built the questionnaire with Questionnaire Star, and the first question required respondents to have used health apps or to have some knowledge of health apps. The questions could be answered on a scale, and every question consisted of a set of statements. Each statement had nine responses, ranging from 1 to 9 according to the evaluation of the degree of the effect, ranging from very unimportant to very important. The questionnaire included basic information (sex, age, education level, and whether they had used or known about fitness apps) and the evaluation of the importance of relevant factors influencing their use.

## 4. Statistics and Analysis

We distributed 420 questionnaires using Questionnaire Star to adults aged 18–65 years. The first part of the questionnaire asked the respondents whether they know about or have used a fitness app; if they responded yes, they continued to the second part containing influencing factor questions; if they responded no, the questionnaire ended. According to the data collected from the questionnaires, 347 out of 420 people had knowledge of or had used a fitness app. Among the 347 questionnaires, those with a shorter filling time and multiple scores of the same response were considered invalid questionnaires and deleted. Of the 347 questionnaires, 310 were valid, with an effective rate of 89.33%. Among them, men and women accounted for 50.32% and 49.68%, respectively, of the respondents, with most being 18–30 years old, followed by 31–40 years old (Table 3). Subsequently, we performed frequency analysis and AHP on the 310-sample data to derive the weight values for each index and perform the consistency test.

### 4.1. Confidence and Validity Analysis

Reliability research methods are often used when analyzing research projects to test whether they are reasonable and meaningful (Table 4). Validity analysis is performed using factor analysis methods to verify the validity level of the data with KMO values of commonality, variance explained values, factor loading coefficient values, and other indicators. KMO values are used to select the suitability of the fact extraction, and commonality values are used to eliminate unreasonable items (Table 5).

#### 4.1.1. Questionnaire Reliability Test

The reliability coefficient, Cronbach’s alpha, is used to measure the internal consistency or reliability of an instrument or questionnaire. This coefficient is often used for questionnaires developed using multiple Likert scales to determine whether the scale is reliable. We used Cronbach’s alpha to determine the reliability using SPSSAU, resulting in a Cronbach’s alpha value of 0.917 (Table 4), which indicated the good reliability and high internal consistency of this questionnaire for additional analysis.

#### 4.1.2. Questionnaire Validity Test

Validity testing involves the measurement of the validity of the questionnaire research data: whether the results obtained through the questionnaire are true and whether the respondents’ evaluations are objective. For questionnaire validity tests, structural validity is used, and the results reflect the accuracy of the questionnaire items. Structural validity reflects the relationship between the questionnaire measurement results and the measured items. The two indicators of structural validity are the KMO and Bartlett’s sphericity test. The coefficients of the KMO range from 0 to 1; the closer the coefficient is to one, the higher the validity of the questionnaire. Bartlett’s sphericity test result needs to be less than 0.01. We imported the questionnaire into SPSSAU for analysis, finding a KMO value of 0.926 and Bartlett’s sphericity test result of 0.000, which indicated that the structural validity of the questionnaire was excellent and all the factors had a strong correlation (Table 5). According to Bartlett’s sphericity check, the significance of this check is infinitely close to zero. Therefore, the questionnaire has appropriate validity and meets the conditions of applicability for factor analysis.

### 4.2. Determination of Index System Weights Based on Hierarchical Analysis

#### 4.2.1. Establishing Comparison Judgment Matrix

Based on the evaluation scales in the AHP, the elements in the product hierarchy model are compared and assigned. To use mathematical methods for data processing, the data needs to be transformed into a matrix to quantify the results and determine the importance of the design elements. Supposing n influencing elements, *b*_1_ ..., *b_i_* ..., *b_j_* ..., *b_n_*, the project elements are compared with each other in pairs and transformed into a judgment matrix as follows:

(1) $$B=\left[\begin{array}{c} 1\cdots b_{1i}\cdots b_{1j}\cdots b_{1n}\\ b_{i1}\cdots 1\cdots b_{ij}\cdots b_{in}\\ b_{j1}\cdots b_{ji}\cdots 1\cdots b_{jn}\\ b_{nl}\cdots b_{ni}\cdots b_{nj}\cdots 1 \end{array}\right]={\left(b_{ij}\right)}_{nxn}$$

The Perron–Fresenius theorem shows that matrix *B* has a unique nonzero eigenroot, i.e., the largest eigenroot $\left(\lambda_{max}\right)$ corresponds to the eigenvector (w).

(2) $$B_{w}=\lambda_{max}w$$

The specific steps for calculating the feature vectors using the sum-product method are as follows:

Normalize the data in *b* by column.

(3) $$\bar{b_{ij}}=b_{ij}/\sum_{j=1}^{n}\bar{b_{ij}}\left(i,j,\ldots ,n\right)$$

Sum the normalized matrix peers.

(4) $$\tilde{w_{i}}=\sum_{j=1}^{n}\bar{b_{ij}}\left(i=1,2,\ldots ,n\right)$$

Divide the summed vector by n to obtain the weight vector.

(5) $$\tilde{w_{i}}=\tilde{w_{i}}/n$$

Find the maximum characteristic root.

(6) $$\lambda_{max}=\frac{1}{n}\sum_{i=1}^{n}\frac{n}{i=1}\frac{{\left(B_{w}\right)}_{i}}{w_{i}}$$

where ${\left(B_{w}\right)}_{i}$ denotes its component of the vector $B_{w}$.

Based on the above Equations (1)–(6), we calculated the weight values of the designed element objectives at the criterion and program levels and then ranked them in terms of importance to complete the decision on the influencing factors.

#### 4.2.2. Calculating Weight Coefficients

Because the hierarchical structure model we constructed had more elements at the program level and the generated judgment matrix order was greater than nine, we used a combination of the AHP and entropy methods for data processing. We formed the evaluation indexes by decomposing the problem and comparing the judgment. We calculated the weightings to obtain the comprehensive weight values of the elements at the program level. We first calculated the AHP-based weight.

Check the consistency of matrix *B*. Calculate:

(7) CR=CI/RI

where CI is the consistency index; CR is the consistency ratio; and RI is the common random consistency index.

(8) $$\mathrm{CI}=\left(\lambda_{max}-n\right)/\left(n-1\right)$$

From Equation (8), CR can be calculated. CR < 0.1 indicates that the calculation of matrix B is qualified and valid. If CR > 0.1, the matrix needs to be corrected [52].

Based on the above-mentioned ideas, we constructed the judgment matrix and calculated the weights of the impact factor (Table 6, Table 7, Table 8, Table 9 and Table 10).

From the outcomes in Table 6, Table 7, Table 8, Table 9 and Table 10, we found that the CR values of the judgment matrices were all <0.1, so we skipped the consistency test. From this, we calculated the weighting for the program-level elements to obtain the comprehensive weight values of the program-level elements (Table 11).

#### 4.2.3. Consistency Test

We performed consistency tests on the combined weight values of all the design elements in Table 11, and the operational procedure and results are shown:

(9) $$CI=\sum_{j=1}^{m}b_{j}CI_{j}=(0.000\;0.000\;0.000\;0.000)\left(\begin{array}{c} 0.2667\\ 0.2759\\ 0.1909\\ 0.2663 \end{array}\right)=0$$

(10) CR=CI/RI=0÷1.520=0<0.1

Based on Equations (9) and (10), CR = 0 < 0.1. The hierarchical total ranking of matrix B was consistent with the consistency test principle, and we found that the calculations of the comprehensive weight values of the scheme-level elements in Table 9 were scientific and reasonable and so could effectively guide the practical analysis [53].

### 4.3. Entropy Method Weights

The entropy approach is a goal-undertaking method, and the weights determined with this method are more accurate than those obtained with the subjective challenge method. Entropy is a measure of the disorder of a system, and by measuring the degree of disorder in the variables, the weights of indicator variables can be obtained by comparing the amount of information possessed by the variables. However, the method is prone to imbalanced weights due to the large dispersion of a certain indicator.

In the entropy weight approach, the entropy weight of the index is first calculated by applying the record’s entropy after standardizing the authentic data. The rank of item *X*, when the index is positive, is standardized with the following system.

(11) $$Y_{ij}=\frac{X_{ij}-X_{i_{min}}}{X_{i_{max}}-X_{i_{min}}}$$

When the indicator is negative, its normalization treatment formula is:

(12) $$Y_{ij}=\frac{X_{i_{max}}-X_{ij}}{X_{i_{max}}-X_{i_{min}}}$$

where $X_{imax}$ and $X_{imin}$ are the maximum and minimum values of the indices, respectively; *Y_ij_* is the normalized result setting of the first impact factor affecting prevention and control. For a certain impact factor *j*, its information entropy calculation formula $E_{j}$ is:

(13) $$E_{j}=-\frac{1}{\ln m}\sum_{i=1}^{m}P_{ij}\ln P_{ij}$$

(14) $$P_{ij}=\frac{Y_{ij}}{\sum_{i=1}^{m}Y_{ij}}$$

where *P_ij_* is the proportion of the standardized value and *Y_ij_* is the total standardized value. If the information entropy *E_j_* of the factor influencing prevention and control is smaller, the degree of variability in the factor is smaller, the sample data are more orderly, the differentiation ability of the evaluation object is larger, and the information utility value provided by the factor is larger. The stronger the influence on border prevention and control, the higher the weight; conversely, the larger the information entropy *E*, the larger the degree of variability is for the influence factor, and the information utility value provided by the factor and the weight is smaller.

According to the calculated information entropy of each factor,$\;E_{1}$, $E_{2}$,⋯,$E_{k}$, the weight formula $W_{j}$ for each factor can be calculated as follows:

(15) $$W_{j}=\frac{1-E_{j}}{k-\sum_{j=1}^{k}E_{j}}$$

Based on Equations (11)–(15), we calculated the weights of each index (Table 12 and Table 13).

### 4.4. Integrated Weight Calculation

In this study, based totally on the reliability and availability of the data, we used two strategies (subjective and goal weight replication) to resynthesize and assign the weights of the influencing factors affecting the use of health apps by adults. We continuously revised the influencing elements. The results indicated a large difference in the weighting of the indicators using the two methods, especially in the process of determining the weighted values of indicators H11 and H3. This difference was due to the difference between the weights calculated by the mathematical model and our understanding of the application of the indicators in practice, which led to the difference in the weight coefficients. Our finding also further confirmed the necessity of studying the assignment of subjective and objective integrated weights.

Based on the results of assigning weights to the indicators by the above two methods, we calculated the combined weight $C_{j}$:

(16) $$C_{j}=\frac{w_{i}w_{j}}{\sum_{i=1}^{n}w_{i}w_{j}}$$

where $w_{i}$ and $w_{j}$ represent the weights of the evaluation indexes calculated by the hierarchical analysis and entropy value method, respectively. We synthesized and calculated the results of both the subjective and objective assignments (Table 14 and Table 15).

### 4.5. Data Analysis

The weightings of B1 (PU), B2 (PEOU), B3 (perceived cost), and B4 (nonpublic motivation) for the assessment goal layer A were 0.2808, 0.2565, 0.1997, and 0.2630, respectively. The comprehensive weights of H1, H2, and H3 were 0.0948, 0.0874, and 0.0795, respectively. The weights of H4 (technical grade), H5 (interaction effectiveness), and H6 (system compatibility) for B2 PEOU were 0.0857, 0.0900, and 0.0867, respectively. The weight values of H7 (financial cost) and H8 (privacy cost) for B3 (perceived cost) were 0.0859 and 0.1000, respectively. The weight values of H9 (health concern), H10 (outcome expectations), and H11 (social influence) for B4 (personal motivation) had weight values of 0.0867, 0.0862, and 0.1172, respectively.

**Figure 2:**
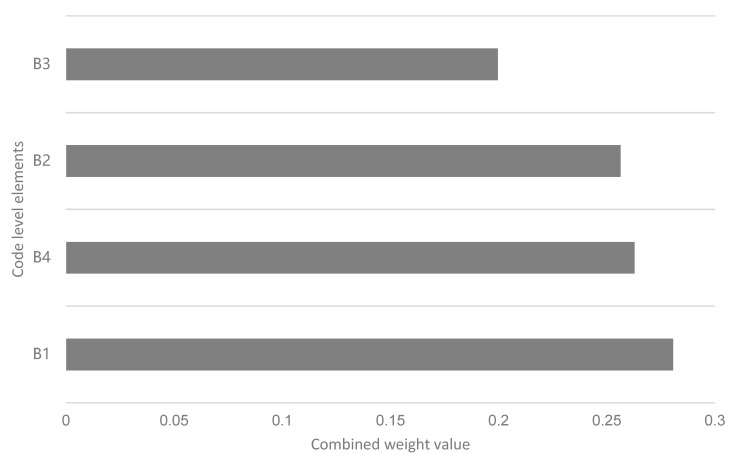
Statistical comprehensive weight values of criterion layer.

**Figure 3:**
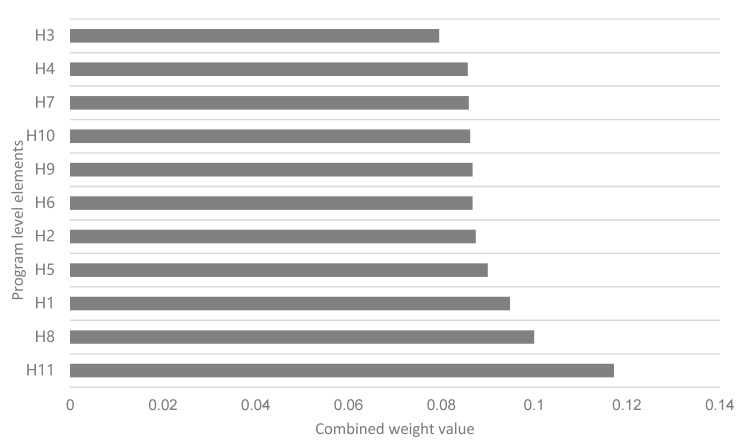
Statistical comprehensive weight values of scheme layer.

According to the criterion-stage weight values, we found that the ranking of the elements influencing the use of health apps by adults under the impact of COVID-19 were B1 (PU), B4 (nonpublic motivation), B2 (PEOU), and B3 (perceived cost) (Figure 2). That is, for this group, PU ranked first when people chose or used fitness apps, accompanied by private motivation, which was especially influential, and then the PEOU and perceived value. According to the weight values of the scheme layer, we found that these adults were more influenced by H11 (social), H8 (privacy cost), H1 (content adaptability), H5 (interaction effectiveness), and H2 (content relevance), and less influenced by H6 (system compatibility), H9 (health concerns), H10 (outcome expectation), H7 (financial cost), H4 (technical grade), and H3 (content quality) (Figure 3).

## 5. Discussion

First, PU had the most notable effect on the adult use of fitness apps. The indicators we used to measure B1 (PU) were the pair of H1 (content adaptability) and H2 (content relevance), as well as H3 (content quality). Among them, H1 was the influencing factor with the highest weight because the users of fitness apps are of different ages and have different exercise purposes, physical bases, and exercise programs, so users have different requirements for the content adaptability of fitness apps. If the app is not based on scientific and effective assessment data for personalized program settings, the user may perform improper or ineffective exercises. For example, some apps directly recommend HIIT exercise programs for primary training; such training is characterized by high exercise intensity, short duration, and high energy consumption, so is not suitable for most primary fitness, leading to the user feedback of exercise intensity being too high and the exercise program being difficult to implement. However, for people experienced with exercise, this kind of exercise may not meet their fitness needs. Exercise apps should also help users avoid injury due to exercise, allowing users to reduce the difficulty of the exercise and to choose low-risk and low-threshold programs to ensure the safety of exercise; however, this may prevent users from achieving the purpose of the exercise.

Second, personal motivation considerably influenced the study group’s intention to use fitness apps. The indicators measuring B4 (personal motivation) were H9 (health concerns), H10 (outcome expectation), and H11 (social influence). Among them, H11 and H9 had higher weights. The higher weight of H11 indicated that people were more influenced by their community when using fitness apps. Social impact refers to the stress and impact that people experience from the humans around them when they perform a behavior [52]. The environment and people around an individual, such as family environment and members, friends, work environment, colleagues, etc., can substantially influence their specific behavior. Community influence is more important in Chinese culture. If companies want to improve their social influence, a long-term process is required; they should implement measures to proactively improve the quality of their products and services, improve customer experience, and assume their social role. H9 had a stronger impact on personal motivation, indicating that the study group was aware of the importance of physical health; therefore, concerns about their health will increase their autonomy in fitness. Given the effect of COVID-19, people’s fitness awareness has increased, and their intention to engage in PA is stronger, increasing their motivation to use health apps.

Third, B2 (PEOU) strongly impacted the study participants’ use of fitness apps. PEOU was influenced in decreasing order by interaction effectiveness, system compatibility, and technical level. This indicated that this group preferred software that was easy to operate, appropriate, with a reasonable design, and that had a user-friendly software interface, which could provide a good user experience. The discovery, installation, and login of the software, the use, recording, and uploading of results and sharing in the fitness process should be easy and not require time or effort to learn. In addition, the interface of the software should be reasonably designed, and the content should be relevant so that the user feels that this product is suitable for them. In the design of mobile fitness apps, user-friendliness should be considered.

Fourth, B3 (perceived cost) had relatively little impact on the use of fitness apps by the study group; however, adults of this age were considerably more worried about H8 (privacy cost) than financial cost according to the weights of the scheme-level indicators. Users face many risks in the process of using the mobile Internet; private information may be leaked, and the perception of privacy risks negatively affects the perceived value. Privacy price has a sizable poor impact on the perceived price. Fitness APPsapps should have a clear, effective, and easy-to-understand security privacy policy. Expense cost also affects the users’ experience of perceived cost during use. The perceived economic cost is the users’ perception of objective costs with a certain subjectivity, and the higher the perceived cost, the lower the perceived value. Therefore, enhancing the best of merchandise and offerings and enhancing the value effectiveness is one of the core aggressive benefits of sports activities and health apps. The degree of satisfaction with the merchandise and offerings immediately determines whether or not customers are inclined to use them continuously, and companies should focus on providing users with high-quality products and services.

## 6. Conclusions

Our results showed that, first, in the criterion layer, the weight of PU was 0.2808, which was much larger than that of the other indicators, indicating that PU most strongly influenced the study group’s use of fitness apps under the influence of COVID-19. Among the criteria that we used for measuring PU, the study group was more concerned about content adaptability. Therefore, developers of health apps need to pay attention to the special traits of users, provide more customized and scientific strategies and content, and select reasonable and scientific fitness programs tailored to users according to their age, occupation, height, weight, personal preferences, etc. Second, the weights of personal motivation and PEOU were 0.2630 and 0.2565, respectively, indicating their stronger impact on the willingness of the study groups to use fitness apps. We recommend that fitness APP developers pay attention to the different characteristics of users and provide more personalized service methods and content, improve the fun of exercise, and reduce the fatigue experienced when users exercise. Third, the perceived cost had the lowest weight of 0.1997, indicating a weaker influence on the group’s use of fitness apps. The data of the indicators measuring the perceived cost showed that the study group was much more worried about privacy than the financial cost, indicating that the group had a strong sense of privacy. Security and privacy policies imply a commitment to users’ personal information. Due to the small operating interface of cell phones and portable devices, companies should proactively and prominently display protection policies so that users can feel the company’s commitment to security and privacy.

Currently, the COVID-19 pandemic is still ongoing. Increasing people’s physical activity during the pandemic to ensure physical and mental health and to improve the well-being of the population remains a difficult task. The data from this study can help subsequent fitness app developers understand user needs and provide an empirical basis for subsequent fitness app development or iterations.

## Figures and Tables

**Table 1:** Index system used for analyzing factors influencing use of fitness apps by adults aged 18–65 years.

Target Layer	Guideline Layer	Program Level	References
A: Study factors influencing use of fitness app by adults under influence of COVID-19	B1: Perceived usefulness	H1: Content Adaptation H2: Content Targeting H3: Content Quality	Davis et al. (1989) [27] Karahanna et al. (1999) [28] Beldad et al. (2010) [29]
	B2: Perceived ease of use	H4: Technical Grade H5: Interaction Effectiveness H6: System Compatibility	Davis et al. (1989) [27] Chang et al. (2021) [31]
	B3: Perceived cost	H7: Financial Cost H8: Privacy Cost	Kwon et al. (2022) [47] Wang et al. (2022) [51] Park et al. (2018) [49]
	B4: Personal motivation	H9: Health Concerns H10: Outcome Expectations H11: Social Impact	Park et al. (2018) [49]

**Table 2:** Description of index conversion questionnaire.

Program-Level Indicator	Problem Description
H1: Content Adaptation	Fitness app can provide different exercise categories of fitness programs for you
H2: Content Targeting	Fitness app can meet your individual needs
H3: Content Quality	Fitness app can provide scientific and professional fitness guidance for you
H4: Technical Grade	Fitness app can be quickly opened on different types of devices
H5: Interaction Effectiveness	Fitness app interface design is clear, convenient, and easy for you to use
H6: System Compatibility	Fitness app can share data with all kinds of your wearable devices
H7: Financial Cost	Fitness app can save you money
H8: Privacy Cost	Fitness app can protect your personal privacy
H9: Health Concerns	Fitness apps can ease your health worries
H10: Outcome Expectations	Fitness app can achieve your expected results
H11: Social Impact	Fitness app has a high social impact on you

**Table 3:** Basic information of the questionnaire respondents.

Variable	Options	Frequency	Percentage (%)
Sex	Male	156	50.323
	Female	154	49.677
Age group (years)	18~30	225	72.581
	31~40	51	16.452
	Under 18	15	4.839
	41~50	12	3.871
	Over 50	7	2.258
Total		310	100.000

**Table 4:** Cronbach reliability analysis.

Cronbach’s α	Standardized Cronbach’s α	Number of Items	Number of Samples
0.917	0.918	11	310

**Table 5:** KMO and Bartlett test results.

KMO Value		0.926
Bartlett’s sphericity test	Approximate cardinality	1857.364
	df	55.000
	P	0.000 ***

Note: *** represents a significance level of 1%.

**Table 6:** Target layer judgment matrix and weight value of influencing factor.

A	B1	B2	B3	B4	$w_{i}$	$\lambda_{max}$	CI	CR
B1	1	0.990	0.972	1.005	0.2709	4.000	0.000	0.000
B2	1.010	1	0.982	1.016	0.2737			
B3	1.029	1.019	1	1.035	0.1858			
B4	0.995	0.984	0.967	1	0.2695			

**Table 7:** PU judgment matrix and weight values.

B1	H1	H2	H3	$w_{i}$	$\lambda_{max}$	CI	CR
H1	1	0.987	0.962	0.3276	3.000	0.000	0.000
H2	1.013	1	0.974	0.3318			
H3	1.040	1.027	1	0.3407			

**Table 8:** PEOU judgment matrix and weight values.

B2	H4	H5	H6	$w_{i}$	$\lambda_{max}$	CI	CR
H4	1	0.979	0.989	0.3297	3.000	0.000	0.000
H5	1.022	1	1.010	0.3369			
H6	1.012	0.990	1	0.3334			

**Table 9:** Perceived cost judgment matrix and weight values.

B3	H7	H8	$w_{i}$	$\lambda_{max}$	CI	CR
H7	1	0.970	0.4924	2.000	0.000	0.000
H8	1.031	1	0.5076			

**Table 10:** Personal motivation judgment matrix and weight values.

B4	H9	H10	H11	$w_{i}$	$\lambda_{max}$	CI	CR
H9	1	0.991	1.074	0.3401	3.000	0.000	0.000
H10	1.009	1	1.083	0.3431			
H11	0.932	0.923	1	0.3168			

**Table 11:** Comprehensive weight values of criterion-layer elements.

Guideline Layer	Guideline-Layer Weights	Program Level	Program-Level Weights
B1	0.2709	H1	0.0888
		H2	0.0899
		H3	0.0922
B2	0.2737	H4	0.0902
		H5	0.0922
		H6	0.0913
B3	0.1858	H7	0.0915
		H8	0.0943
B4	0.2695	H9	0.0917
		H10	0.0924
		H11	0.0854

**Table 12:** Weight results of each criterion layer based on entropy method.

Guideline Layer	Information Entropy Value $E_{j}$	Information Utility Value	Weighting Factor $w_{j}$
B1	0.9963	0.0037	0.2576
B2	0.9966	0.0034	0.2329
B3	0.9961	0.0039	0.2671
B4	0.9965	0.0035	0.2425

**Table 13:** Weight results of each index based on entropy method.

Guideline Layer	Information Entropy Value $E_{j}$	Information Utility Value	Weighting Factor $w_{j}$
H1	0.9946	0.0054	0.0968
H2	0.9951	0.0049	0.0882
H3	0.9957	0.0043	0.0782
H4	0.9952	0.0048	0.0861
H5	0.9951	0.0049	0.0885
H6	0.9952	0.0048	0.0861
H7	0.9953	0.0047	0.0851
H8	0.9947	0.0053	0.0962
H9	0.9952	0.0048	0.0857
H10	0.9953	0.0047	0.0846
H11	0.9931	0.0069	0.1244

**Table 14:** Comprehensive weight results (criterion layer) obtained using two weighting methods.

Guideline Layer	Hierarchical Analysis Weight $w_{i}$	Entropy Method Weight $w_{j}$	Combined Weight $C_{j}$
B1	0.2709	0.2576	0.2808
B2	0.2737	0.2329	0.2565
B3	0.1858	0.2671	0.1997
B4	0.2695	0.2425	0.2630

**Table 15:** Comprehensive weight results (scheme layer) obtained by two weighting methods.

Indicator	Hierarchical Analysis Weight $w_{i}$	Entropy Method Weight $w_{j}$	Combined Weight $C_{j}$
H1	0.0888	0.0968	0.0948
H2	0.0899	0.0882	0.0874
H3	0.0922	0.0782	0.0795
H4	0.0902	0.0861	0.0857
H5	0.0922	0.0885	0.0900
H6	0.0913	0.0861	0.0867
H7	0.0915	0.0851	0.0859
H8	0.0943	0.0962	0.1000
H9	0.0917	0.0857	0.0867
H10	0.0924	0.0846	0.0862
H11	0.0854	0.1244	0.1172

## Data Availability

The experimental data used to support the findings of this study are included in the article.
